# Microscopic Structure of Metal

**Published:** 1881-06

**Authors:** 


### MICROSCOPIC STRUCTURE OF METALS.

Some observations on the minute structure of metals, recently communicated to *Nature* by Mr. J. V. Elsden, are both interesting and instructive. Notwithstanding the great opacity of metals, it is quite possible to procure, by chemical means, metallic leaves sufficiently thin to examine before the microscope by transmitted light. Silver leaf, for example, when mounted upon a glass slip and immersed for a short time in a solution of cyanide of potassium, perchloride of iron, or iron alum, becomes reduced in thickness to any required extent. The structure of silver leaf may also be conveniently examined by converting it into a transparent salt by the action upon it of chlorine, iodine, or bromine. Similar suitable means may also be found for rendering more or less transparent most of the other metals which can be obtained in leaf form. An examination of such metallic sections, says Mr. Elsden, will show two principal types of structure, one being essentially granular and other fibrous. The granular metals, (of which tin may be taken as an example) present the appearance of exceedingly minute grains, each one being perfectly isolated from its neighbor by still smaller interspaces. The cohesion of such leaves is very small. The fibrous metals, on the other hand, such as silver and gold, have a very marked structure. Silver, especially, has the appearance of a mass of fine elongated fibers, which are matted and interlaced in a manner which much resembles hair. In gold, this fibrous structure, though present, is far less marked. The influence of extreme pressure upon gold and silver seems to be, therefore, to develop a definite internal structure. Gold and silver, in fact, appear to behave in some respects like plastic bodies. When forced to spread out in the direction of least resistance their molecules do not move uniformly, but neighboring molecules, having different velocities, glide over one another, causing a pronounced arrangement of particles in straight lines. This development of a fibrous structure, by means of pressure, in a homogeneous substance like silver, is an interesting lesson in experimental geology, which may serve to illustrate the probable origin of the fibrous structure of comparatively homogeneous limestones like those of the Pyrenees, Scotland, and the Tyrol.—*Scientific American.*

